# Untangling the complex web of alcohol policy needs and potential solutions in Brazil: evidence from civil society and political stakeholders

**DOI:** 10.1093/heapol/czaf104

**Published:** 2025-12-18

**Authors:** Inaê Valério, Isabelle Uny, Alejandra Burela, Marina Piazza, Mark Petticrew, Niamh Fitzgerald, Zila M Sanchez

**Affiliations:** Departament of Preventative Medicine, Federal University of São Paulo, São Paulo, Brazil; Institute of Social Marketing and Health (ISMH), Faculty of Health Sciences and Sport, University of Stirling, Stirling FK9 4LA, United Kingdom; Faculty of Public Health and Administration, University Peruana Cayetano Heredia, San Martín de Porres, Peru; Faculty of Public Health and Administration, University Peruana Cayetano Heredia, San Martín de Porres, Peru; London School of Hygiene and Tropical Medicine, London, United Kingdom; Institute of Social Marketing and Health (ISMH), Faculty of Health Sciences and Sport, University of Stirling, Stirling FK9 4LA, United Kingdom; Departament of Preventative Medicine, Federal University of São Paulo, São Paulo, Brazil

**Keywords:** alcohol policy, decision-makers, Brazil, advocacy, best-buys, industry lobbying

## Abstract

Implementing evidence-based alcohol policies can reduce the negative impact of alcohol consumption on public health. However, Brazil has permissive alcohol policies and weakly adheres to World Health Organization’s recommendations as the ‘best buys’. To explore stakeholders’ perceptions of alcohol policy needs and barriers in Brazil, we conducted semi-structured interviews with 31 stakeholders, including 15 from civil society and 16 policymakers. Civil society participants included non-governmental organization leaders addressing alcohol-related issues, while policymakers comprised civil servants and politicians experienced in alcohol-related harms. Interviews were transcribed verbatim and thematically analyzed using a deductive approach guided by research questions and an inductive approach to identify emergent themes. Most participants supported World Health Organization-recommended ‘best buy’ policies regulating alcohol's marketing. However, agreement on price and availability control was not unanimous. All participants acknowledged significant political barriers to adopting these policies, including intentional delays in parliamentary voting, industry lobbying, and arguments about infringing on rights such as freedom. Facing obstacles to advancing population-level policies, stakeholders often shifted their focus to individual-level interventions, such as education and treatment. While these were recognized as less effective, educational efforts were highlighted for raising public awareness of alcohol’s harms and changing normative beliefs. Participants noted the lack of a formal coalition to reduce alcohol-related harm, despite its perceived necessity. Overall, stakeholders supported population-level alcohol policies but were pessimistic about their implementation due to political barriers. Many, particularly from civil society, emphasized small-scale, targeted interventions as a more feasible alternative to address alcohol-related harm in Brazil.

Key messages

- Stakeholders highlight the alcohol industry’s lobbying and marketing efforts to normalize consumption and resist regulation, using economic arguments to shape legislation and delay evidence-based policies.
- Although there is broad support for regulating marketing, availability, and pricing, pessimism remains about implementation due to industry opposition, political resistance, and cultural barriers. Educational interventions are seen as immediate but less effective alternatives.
- The absence of a unified coalition advocating evidence-based policies limits progress. Stakeholders stress the need for strategic alliances among civil society, health experts, and policymakers to counteract industry influence and push for legislative change.

## Introduction

Alcohol consumption is a major risk factor for diseases, injuries, and social harms, causing ∼2.6 million deaths annually (WHO 2024). Brazil ranks fourth in Latin America for per capita alcohol consumption, with a rate of 7.7 L per person annually. The country also has the highest prevalence of heavy continuous drinking in South America, with 8% of drinkers engaging in this behavior (WHO 2024). Women with higher education have seen the largest increase in alcohol consumption in recent years (Wendt et al. 2021).

Effective interventions to reduce alcohol-related harms include restrictions on marketing, reducing alcohol sales outlet density, price increases (e.g. taxation), and community mobilization (Babor et al. 2022). Brazil has enacted policies like banning alcohol to minors and setting a zero-blood alcohol limit for drivers, but challenges remain in implementation and policy approval (de Oliveira et al. 2021). The country scores 51.6% on the Alcohol Policy Score, reflecting some alignment with World Health Organization (WHO) recommendations, but the alcohol industry’s political influence hinders progress (Pan American Health Organization 2018, Pinsky et al. 2022).

Despite the evidence of effective tobacco control strategies, alcohol policy advancements in Brazil are limited. Research on alcohol policy processes in low- and middle-income countries, including Brazil, is scarce, with studies mainly focusing on industry interference (Pinsky 2014, Pantani et al. 2017). Stakeholder views and experiences in policymaking are understudied, making it difficult to develop advocacy strategies.

This study aims to fill this gap by exploring Brazilian stakeholders’ perceptions of alcohol’s role in society, harm reduction strategies, and policy solutions. It also aims to support the development of coordinated advocacy strategies and a national coalition to advance alcohol policy in Brazil. Although academics, non-governmental organizations (NGOs), and some parliamentarians are engaged in the issue, their efforts remain fragmented, while the alcohol industry benefits from organized lobbying. By mapping diverse stakeholders, we seek to identify common ground and opportunities for collaboration, providing an evidence base for a unified coalition to counter industry influence and strengthen alcohol control policies.

## Method

This manuscript was written following the recommendations of the Consolidated Criteria For Reporting Qualitative Research (COREQ) for qualitative interviewing and reporting (Tong et al. 2007).

### Research design and recruitment

We conducted a qualitative study based on in-depth, semi-structured interviews among 31 stakeholders. We included national-level stakeholders in Brazil, preferably those with a direct remit concerning alcohol-related harms. Two groups were purposively sampled: (i) policy stakeholders, such as elected politicians and civil servants from diverse departments and levels of seniority, with priority given to those currently engaged in alcohol policy agendas; and (ii) civil society (CS) stakeholders in senior or leadership roles within national health bodies or NGOs/charities addressing issues exacerbated by alcohol consumption (e.g. road safety, violence against women, and cancer). Individuals with conflicts of interest related to the alcohol beverage industry were excluded.

The recruitment strategy for this research was carried out using a pragmatic snowball sampling (Patton 2003). In the initial phase, we used our extensive networks to identify 10 key stakeholders involved in the field, including those publicly known, and those identified by (name removed for anonymization purposes), a recognized expert in alcohol studies in Brazil with a long-standing and diverse network of stakeholder contacts. These stakeholders held relevant positions at NGOs, advocacy coalitions, and as politicians, all publicly known as involved in alcohol issues. Initial contact was established by email, WhatsApp, or by personal telephone.

Then, those 10 key stakeholders indicated a total of 8 other stakeholders who met our criteria. All of them accepted to be interviewed. We also accessed some individuals who had extensive networks with government officials and/or NGOs, but who did not meet our sample inclusion criteria. Through their referrals, we recruited 9 further interviewees. To complete our sample by including other perspectives outside the alcohol field, we used Google search to find organizations that did not necessarily work with alcohol, but worked with populations more vulnerable to alcohol-related harms—such as NGOs that serve the LGBTQIA+ population, people living on the streets, sexual workers, and individuals in social vulnerability, that combat violence against women—and interviewed their representatives. At this stage, 4 individuals were interviewed and completed our final sample. Characterization of the interviewees is presented in Table 1 and describes their professional positions.

**Table 1.** Interviewee characteristics

Participant number	Gender	Years of experience in field of alcohol or drugs	Type of interviewee/current field of work (alcohol/tobacco/other)
**Civil society (*n* = 29)**			
CS02	Male	8	NGO Executive/non-communicable diseases risk factor
CS03	Male	5+	NGO Executive/alcohol
CS07	Female	6+	NGO Executive/tobacco
CS08	Male	13	NGO Executive/substance use
CS12	Male	17	NGO Executive/drink driving
CS13	Female	15	Community Coalition NGO
CS14	Male	11+	Advocacy for marginalized communities
CS15	Male	6+	NGO staff/projects of support for drug users
CS17	Female	11	NGO staff/harm reduction
CS18	Female	0	Women’s support NGO
CS20	Female	5+	People with disability NGO
CS22	Female	25	NGO staff/harm reduction
CS24	Female	17	NGO staff/drug user care
C25	Male	25	Addiction treatment NGO
CS27	Male	24	Religious NGO
**Civil servants, policymakers, and** **legislators (*n* = 31)**			
PS1	Female	1	Former representative of the Ministry of Health
LEG/PS4	Male	10+	Prosecutor
PS5	Female	5–10	Former representative of the Ministry of Economy
LEG/PS6	Male	11	Prosecutor
LEG/PS9	Male	20+	City councilor
PS10	Female	17	Representative of the Ministry of Social Development
PS11	Male	0	Former representative of the Ministry of Health
PS16	Male	0	Lieutenant
PS19	Male	20+	Former representative of the Ministry of Justice
LEG21	Female	8	Federal Deputy
PS23	Male	20+	Municipal health secretary
LEG26	Male	0	City councilor
LEG28	Female	4+	State Deputy
PS29	Female	4+	Representative of the National Institute of Cancer
PS30	Male	4	Senator’s assistant
LEG31	Male	30+	Federal Deputy

### Data collection

Interviews were conducted by three PhD researchers between March 2022 and February 2023. Participants took part in an in-depth, audio-recorded, interview lasting on average 60 min, mostly on Zoom, with one face-to-face at interviewee’s request. We used a semi-structured interview guide which covered: (i) perceptions of alcohol consumption and related local culture; (ii) perceived level of harms; (iii) factors underpinning types of alcohol-related harm most relevant to their organization or remit, including age, gender, or equity; (iv) policy solutions and priorities for action; (v) awareness of alternative alcohol problem/policy narratives; (vi) perceptions and opinions about WHO best-buys alcohol policies (marketing, availability, and prices); (vii) experience of advocacy/lobbying by interested parties; (viii) current/past action, interests/views regarding advocacy or coalition building; and (ix) any other factors perceived as relevant to policy progress.

After data collection, the audio files in Portuguese were transcribed verbatim using the transcription software dictation.io/speech. Subsequently, researchers manually corrected and completed the transcription to ensure quality and veracity. Transcripts in the native language (in Portuguese) were then anonymized and imported into NVivo 12 for analysis. We adopted a framework approach (using framework matrices in NVivo) to summarize (in Portuguese) and analyze the coded data (Smith and Firth 2011, Gale et al. 2013, Hackett and Strickland 2019). To develop a coding framework, our team engaged in multiple discussions, incorporating both deductive (guided by the research questions and topic guide) and inductive (derived from the interview data) strategies. This draft framework was initially tested on three transcripts, then refined before being applied it to the remaining interviews using NVivo software.

### Data analysis

Initially, we conducted thematic analysis of the transcripts using the approach proposed by Braun and Clarke (2024). Once this coding was completed, we summarized the data under each theme. These summaries were further developed into interpretive memos of the themes (seeking to identify the links between them), which led to the first draft of the results. Details of the coding framework and interpretive memos are available in the online supplementary material. (Name removed for anonymization purposes) and the rest of the research team met on several occasions to discuss the results and their interpretation (Braun and Clarke 2024).

We used ChatGPT (OpenAI, GPT-5) to assist with the translation of text passages from Portuguese to English. All outputs were reviewed and edited by the authors for accuracy and clarity.

## Results

A total of 31 individuals, 18 men and 13 women, participated in the study (see Table 1). Among them, 16 were legislators (LEG) or public servants (PS), and 15 were affiliated with CS institutions. For those who had specific experience or were closely involved with alcohol policy, their experience varied from 4 to 25 years (Table 1). We identified six categories (each with a number of sub-themes) in our analysis: (i) normalization of alcohol use in Brazilian culture; (ii) the role of the alcohol industry; (iii) perceptions of current policies; (iv) the role of parliament; (v) perceptions of best-buys, and (vi) proposed solutions.

### Normalization: is alcohol indissociable from Brazilian culture?

#### Lack of general population awareness

All participants identified the normalization of alcohol consumption in Brazil as a major obstacle to the implementation of public policies. They noted that alcohol is often viewed as an integral part of the Brazilian lifestyle, ubiquitous at celebrations and social gatherings. The majority of participants attributed this normalization and minimization of alcohol use, in part, to a general lack of awareness about the harms associated with alcohol consumption. Some mentioned that since alcohol can be legally purchased, it is not commonly recognized as a drug. This normalization is further exemplified by the tendency of some family members to encourage children to try alcoholic beverages, as highlighted by one participant.

‘Sometimes the child is there, and the person puts the pacifier in the caipirinha [a Brazilian mixed drink made with cachaça and lemon juice] or beer and puts it in the child's mouth.’ (LEG/PS09)

Most participants noted that, although the public recognizes the harms of alcohol in situations such as drink driving or violent behavior, they often underestimate the risks of low-dose consumption, implying the existence of a ‘safe’ level of intake. Moreover, most participants, whether CS representatives, policymakers, or LEG, felt that the public lacks awareness of the chronic damage caused by alcohol, even at lower consumption levels, resulting in a predominant focus on short-term harms.

‘Look, Brazil's main substance of concern is alcohol. Even occasional use of alcohol can have very serious consequences for people and others. Whether it is because there is also a very mistaken perception about the harmful potential of alcohol.’ (PS19)

Most interviewees conveyed a widespread perception that healthcare professionals also have limited understanding of the detrimental impacts of alcohol, including knowledge about the development of alcohol dependence and effective treatment approaches. Consequently, individuals with alcohol-use disorders face stigma, while the belief in the health benefits of moderate alcohol consumption, especially wine, persists, leading people to include it in their diet for health reasons. One participant described efforts to better inform health professionals via training.

‘And with alcohol, we (Ministry) try to demystify it a bit because there used to be the recommendation of [consuming] a glass of wine for cardiovascular diseases. And we always try to delve a little deeper into this (with the health professionals), that for cancer prevention there is no safe dose, so we talk a lot about that’ [PS29]

#### Overemphasis on the crack cocaine problem

Some interviewees observed that the media often sensationalizes the fight against crack cocaine, capitalizing on the contentious nature of the issue in Brazil to attract readers. In contrast, alcohol, which is legal and socially accepted, is portrayed differently. As a result, there is a disproportionate focus on combating illegal drugs, a situation that is intensified by the prevailing ‘war on drugs’ policy, which diverts attention and resources away from alcohol-related policies.

‘Because the government was focused on combating crack, which epidemiologically speaking, is … consumption is 10–11 times lower than alcohol. [..] First, because it is media-driven. Because people addicted to crack, you know, are more visible on the street. It facilitates the discussion for funds to get these people off the street, which has a lot to do with the high financing of Therapeutic Communities (TCs) [residential programs where residents support each other’s recovery] to the detriment of funding community-based services like the CAPS [Public Psychosocial Care Centers].’ [CS17]

#### Festivities and events

Brazilian culture embraces festivities where alcohol consumption is a central element, such as Carnival, which some interviewees referred to as the ‘beer festival.’ Several interviewees expressed that open-bar parties, which are very common throughout Brazil, are the most harmful occasions for alcohol consumption in the country. These parties are events that offer unlimited alcohol for a low entry fee and are frequently attended by adolescents and young adults.

‘Another thing that we were adamant about is the issue of banning open-bar parties. It’s part of Brazilian culture. […] I think that if a person is in an environment that is not open-bar, they will consume half of what they would consume in an open-bar. There is an adrenaline rush that people get from feeling like they are consuming more than what they paid for’. [PS06]

Some interviewees mentioned football games as another huge cultural activity promoting alcohol consumption, given that football is one of Brazil’s main national sports. They mentioned it is common for the beer industry to sponsor sporting events, yet the population seems oblivious to the exposure of children and young people to alcohol advertisements at these venues or on TV, and they seldom question this practice. Alarmingly, according to some stakeholders, a significant proportion of these advertisements aired during sporting events convey a misleading message that alcohol consumption is healthy and even appropriate in sports practices.

### Role of the alcohol industry: power, manipulation, and deception

#### Industry pressure on Congress

Many interviewees depicted the strong influence of the multinational pro-alcohol lobby within the Brazilian legislature, especially emphasizing the dominance of the Brazilian beer industry, which they reported to be the largest globally. Some interviewees, with extensive experience in the legislative process, detailed the strategies employed by large multinationals, highlighting their significant financial resources and powerful political connections, which are actively used to influence public policies to their advantage.

‘So, this lobbying happens because they [alcohol industry] have a lot of resources. They live there in Congress blocking all the laws, as seen in that advertising one that equates beer with water and Coca-Cola, so nothing ever gets through. There are thousands of bills (PL) that have been there for 200 years and never pass because they [the industry] are there every day; they have influence.’ [PS01]

One interviewee, a public servant with experience of working with elected politicians, astutely observed that not only is the alcohol industry tremendously influential, but it also benefits from the backing of an even larger entity, the ‘National Confederation of Industry’, the main representative entity of Brazilian industry. This substantial support shields the alcohol industry from scrutiny and grants it undue protection within the legislative process, raising concerns about potential biases and favoritism.

#### The industry’s influence on the population and perceived societal benefits

Most participants perceived the alcohol beverage industry’s marketing as contributing to the normalization of alcohol consumption. They noted that beer advertising is exempt from Law No. 9294/96 (regulating alcohol television and mass media advertising), allowing beer adverts to be broadcast anytime and anywhere:

‘Legally speaking, there is a provision in the law that prohibits alcohol advertising during prime time, and then there is a provision that says ‘unless it is a beverage of 13% alcohol by volume or below.’ So, beer is not alcohol. Is it [because it is below that content]? So, when you see this, there’s no point in fighting against this system.’ [CS08]

Some participants noted that the alcohol industry maintains its influence on the public by employing market segmentation techniques to tailor advertising for specific consumer groups, fostering a sense of product belonging and maintaining consumer loyalty. They cited the distribution of mixed drinks and attractive packaging as examples aimed at attracting young people. A few participants were concerned about the replacement of the wording on beer cans and bottles. Previously, ‘Drink with moderation’ served as a harm-reduction strategy to prevent excessive consumption, and now has been replaced with ‘Drink wisely’. One interviewee believed this is a strategy to encourage consumption since wisdom connotes positivity and desirability.

‘The issue of public marketing harassment that children and teenagers receive through the invasion in their homes, tablets, cell phones, and previously on TV. If you look at 7 in the morning, it seems like a philosophy class dictated by an advertiser “drink wisely!” it’s almost Aristotelian.’ [PS/LEG04]

Other participants pointed out that alcohol multinationals also extend their influence through corporate social responsibility (CSR) programs. They mentioned that this outward appearance of benevolence can create a positive impression for the general public and politicians. For instance, as explained by a CS participant:

‘Today they sponsor institutes, NGOs, etc., that they also sponsor as a veneer of social responsibility. […] They create strategies for a cause. ‘Oh, I'm combating poverty,’ ‘Oh, donation to the Ambev Institute.'’ [CS14]

A few interviewees felt that the alcohol industry bears some responsibility for alcohol-related harms, and therefore saw it as a contradiction that they also participate in the creation of legislation and campaigns to prevent the abusive use of alcohol and drinking and driving.

Almost all participants perceived that certain politicians resist supporting alcohol consumption regulation policies because they have concerns that such policies could harm tax revenue and job creation by undermining the alcohol industry. Participants noted that these concerns echo arguments used by the alcohol industry.

‘Politicians who block [the approval of alcohol regulation], right!? […] from the argument that this affects job and income generation… job creation. So sometimes there is this kind of debate. Then there is a tax debate about how these companies contribute to national development by paying taxes and so on.’ [CS14]

Additionally, interviewees noted that lawmakers generally have little knowledge about public spending on addressing harms caused by alcohol, which may contribute to the government’s inaction in this area. They emphasized the need to provide objective and robust data on the cost of alcohol to society, in order to educate and convince lawmakers about its economic impact on the economy and public spending.

‘I think we need to sensitize primarily the legislative branch, regarding this issue of cost; ‘look, it costs *x* to drink (including all the rhetoric they talk about, the industry, about how much they earn or how many jobs they provide)’, and ‘it costs *y* to the public coffers (in terms of retirement, default, reduced productivity, etc.)’.’ [PS01]

### Role of Parliament: weak and easily influenced

Most participants believed that Parliament's reluctance to enact alcohol control regulation and legislation stems from concerns about displeasing the population, potentially losing votes, or losing financial support from the alcohol industry in election campaigns.

‘You need to have support in Congress to pass this type of law [alcohol regulation]. I don’t see that happening anytime soon, because it depends on the votes of people who are elected in a system that relies on campaign financing, which is very difficult to change. People have no idea about the conflict of interest. The fact that someone is financed by the alcohol industry and then votes on an alcohol-related bill.’ [PS05]

As a result, according to some interviewees, politicians have not allocated parliamentary funds for prevention, harm reduction, or alcohol treatment intervention programs, but rather to TCs.

‘…there is a lot to do with the high funding of therapeutic communities at the expense of community-based services like CAPS […] So you have a freezing of funding for CAPS, which nowadays work in precarious conditions, without resources, and you have… the first call for funding of therapeutic communities was for R$157 million [30 million Int$].’ [CS17]

Although some interviewees had favorable opinions about TCs, others criticized these structures, arguing that they are ineffective, offer treatment through religious practices, and, above all, receive significant financial support from parliamentarians compared to other sectors that do not have funding.

### Perceptions on current policies: achievements and gaps

#### Existing laws

According to almost all the interviewees, the public policies related to alcohol consumption in Brazil have strengths and weaknesses. One of the strong laws indicated is Law No. 11.705/2008, popularly known as the ‘Dry Law,’ a milestone in Brazilian legislation combating drink driving. However, from those interviewees, all have objections about the application of the law. The lack of adequate enforcement was frequently criticized, as well as the insufficient sanctions applied for drink driving offences.

‘Look at what we can’t achieve: drank, drove, hit someone… paid bail and is now back on streets. Is there anything more indecent than that?’ [PS10]

Most participants also perceived positively the prohibition of selling alcoholic beverages to minors <18 years old (Law No. 13.106/2015). However, they note weaknesses in its implementation, as exemplified by one of the interviewees (at the municipal level):

‘There is actually an orchestrated system. […] And I was contacted. I know someone very close who lives in this square and said ‘there are minors coming from school and consuming alcohol at X bar.’ And we [city hall] went after them, called the Security Council, etc… and every time there was a complaint, when the inspection arrived, the minors were no longer inside the bar; they were no longer close to the beer.’ [LEG26]

#### Progress of tobacco policies compared to alcohol policies

Many interviewees emphasized the significant progress on tobacco control since the 1980s, as compared to alcohol control policies. They speculated that this progress is due to factors such as the tobacco industry lacking the same political and economic power as the alcohol industry, pressure from the international awareness movement about the harms of smoking, and society’s perception of the discomfort caused by passive smoking.

‘First, we need to advance our regulation. Regulation is key… especially regarding alcohol. Having regulation, having warnings on bottles like they do on cigarettes. We need to [implement]… that it causes accidents, it causes death; that needs to be put in place.’ (CS14)

#### Vetoed laws and lengthy processes of approval for alcohol regulations

Some interviewees highlighted that numerous proposed laws related to alcohol consumption face considerable delays in Parliament due to internal procedural obstacles. These barriers include extended review periods, unforeseen voting sessions, or the bundling of unrelated legislative proposals. Those participants felt this leads to a slow legislative process, where many bills are neither voted on nor passed. For example, one interviewee cited a bill intended to regulate alcohol advertising. This bill’s progress was impeded when it was combined with two other unrelated bills, thereby complicating and obstructing its passage through the Senate

### Perceptions on WHO ‘best buys’: beliefs and disbeliefs

WHO ‘best buys’ are cost-effective interventions aimed at reducing non-communicable diseases (WHO 2017). For alcohol control, these include increasing taxes, restricting availability, and limiting advertising to reduce consumption and related harm (WHO 2017).

#### Prices

There was no consensus of opinion amongst interviewees regarding the approval of policies to increase the price of alcoholic beverages. Support for such measures came mainly from some experts in our sample familiar with the SAFER strategy, as well as from stakeholders with strong academic backgrounds, including those holding doctoral degrees in medicine and other disciplines. Those participants argued that alcohol in Brazil is very cheap, which contributes to its excessive use, especially among young people.

‘Here they [young people] drink this thing called ‘Corote’ [flavoured vodka mixture], which is a small drink that costs about R$1. It’s very cheap. Maybe what I think is important is something like increasing the tariffs, because this financial restriction ends up restricting access.’ [CS09]

However, some interviewees believed that even if alcohol became more expensive, people would still buy it or resort to producing their own, boosting the illegal market. A few interviewees argued that the measure would primarily affect the poor, while the wealthy would continue to consume alcohol. Interestingly, advocates of harm reduction and proponents of complete abstinence as a therapeutic strategy, although from seemingly opposing ideological groups, shared similar views on alcohol taxation.

‘We can’t just say, ‘let’s increase the tax and people will stop drinking.’ They won’t. They’ll start making it at home. Brazilians are smart.’ [PS10]

Both supporters and opponents of price increases stated that this measure would never be approved by Congress due to the lack of popular support and the strength of the industry influence.

‘None of them (best-buys) will be accepted, but if you really tax more and increase the price, I still think it would be better. Unfortunately, none of them will pass. Because there will always be the other side that will come in with money. We would need to unite. The population, which is the vast majority, should do that.’ [CS27]

#### Availability

A large number of the interviewees suggested that controlling the availability of alcohol for retail sales is an important measure and particularly emphasized the need to reduce access for minors <18 years of age. Some participants supported reducing alcohol sales hours, citing Spain as an example. However, three believed this would be ineffective, either because businesses wouldn’t comply with the law or consumers would stockpile alcohol at home. Others suggested reducing alcohol density in stores, noting that alcohol is often placed throughout supermarkets alongside food. They proposed limiting availability by designating specific outlets for alcohol sales, similar to liquor stores in the United States and Canada. Although several were concerned that reducing the availability of alcohol in the general population could generate popular discontent and, consequently, discontent with Parliament, the interviewees seem to be aware that reducing access would result in a decrease in alcohol consumption. Interviewees expressed concerns about the increased availability of alcoholic beverages in Brazil, including open-bar parties frequented by teenagers and young adults, and delivery apps. The latter were felt to have made alcohol purchases more accessible, raising concerns about sales to minors and excessive consumption.

‘The purchase of alcoholic beverages has become increasingly accessible, everything is virtual, without any contact or monitoring.’ [CS07]

#### Marketing

The majority of interviewees agreed that, although advancing the control of alcohol marketing in the National Congress faces difficulties, regulating advertising is necessary to reduce the harms of excessive alcohol consumption. Almost all interviewees cited the ban on cigarette advertising and the subsequent reduction in consumption of those products as a comparison.

‘I remember when I was a child…they banned cigarette ads, and I have the perception, at least from cases within my family, that smoking seems to have decreased a lot in Brazil.’ [LEG26]

One paradox highlighted by some participants is the prohibition of alcohol consumption in football stadiums, yet advertising for these drinks is prevalent at these events. They also observed that the alcohol industry’s self-regulation has proven ineffective in controlling advertising. Consequently, stakeholders stressed the urgent need for a strategy to persuade lawmakers and diminish the influence of the industry and media companies in debates concerning alcohol advertising regulation in Brazil. One of the participants suggested a step-by-step approach to make alcohol a matter of concern for politicians and potentially lead to the approval of alcohol regulatory laws:

‘The number one path is public opinion. Secondly, it would be the Executive Branch. Information [technical information about health system expenditures due to alcohol use] coming from the Executive, within Ministries, and government agencies, would also have a great impact. Thirdly: this grassroots work, this work of visiting everyone [the politicians], bringing information [about the harmful consequences of alcohol use].’ [PS30]

### Proposed solutions: from individual strategies to public polices

Broadly speaking our participants tended to fall into two distinct camps on this matter: those advocating the implementation of public health policies at a national level were more commonly experts, while those who favored individual-level interventions over public health policies were more commonly NGO representatives. It is important to note that within the group that believed in individually based solutions, there were some who saw this approach as an alternative way of addressing the problem of alcohol consumption, since currently, more structural changes, such as policies, would not be approved by Congress.

#### Legislative changes

Those of our interviewees who believed in legislative changes proposed: (i) the prevention of early alcohol consumption by raising the legal age to 21 years and installing cash registers that pause when an alcoholic product is scanned, prompting the cashier to verify and enter the customer’s date of birth; (ii) restricting access to bars for domestic violence offenders; and (iii) labeling alcoholic beverages with the effects of alcohol to raise awareness among consumers about the negative health impact of alcohol.

Only one participant expressed an opposing view. She highlighted bartender and market seller training, including requesting identification for the purchase of alcoholic beverages, as successful industry programs. The participant believed that collaboration with alcohol companies could be beneficial in developing alcohol control and prevention strategies.

#### Individual changes

Some participants believed that individual educational activities could serve as a temporary solution while waiting for structural changes, by educating the public about the harms of alcohol in an effort to reduce consumption. Among the specific educational actions proposed were: (i) implementing educational initiatives in schools and promoting campaigns in healthcare services, in collaboration with administrators and professionals in the field; and (ii) providing harm-reduction information to individuals already using substances.

Some participants also expressed that an educated and empowered population could pressure and influence lawmakers to pass legislation.

‘The number one way is public opinion. The National Congress is driven by public opinion. That’s what ultimately matters to them. […] Because if there was a contrary public opinion, it would also reduce consumption, reducing their economic power.’ [PS30]

#### Alternative proposals

Another proposition by a few participants is to increase the collaboration between government, the private sector, and CS groups to address and plan legislative changes related to alcohol consumption in order to unite and leverage resources. Additionally, it was suggested that experts should create a lobbying group that could advocate for stronger alcohol harm-reduction policies to counter the alcohol industry lobbyists in Parliament. Such work could be conducted by experienced professionals capable of presenting effective solutions to the problem and presenting legislative proposals and information in a simplified way.

‘[…] we will really need a coalition of third-sector organizations, government, academia, companies, and eventually different groups to partner with those we currently don’t partner with to address a very strong cultural change on this risk factor (alcohol).’ [CS02]

It is important to mention that it was not clear what companies this interviewee felt would be interested in working to reduce alcohol harm.

## Discussion

This study explored stakeholders’ perceptions of alcohol’s role in Brazilian society, strategies to reduce harms, and policy solutions, along with their experiences in policymaking. Findings highlight the normalization of alcohol consumption in Brazilian culture and the significant influence of the alcohol industry over Parliament and politicians’ interests. Stakeholders unanimously supported regulating alcohol advertising. Regarding availability, many endorsed limiting access by reducing sales hours and restricting sales to specific stores, though some believed these measures could be circumvented. Pricing strategies revealed no consensus: some advocated for taxation, while others argued it might be ineffective or exacerbate inequalities. Raising the legal drinking age and implementing individualized educational initiatives, such as increasing awareness of alcohol-related harms in schools and healthcare services, also emerged as potential solutions.

The difficulty in approving effective alcohol regulations is not unique to Brazil. A systematic review of alcohol policies in Latin America and the Caribbean (LAC) highlights low political commitment to treating alcohol as a public health priority (Medina-Mora et al. 2021). The alcohol industry’s influence, through campaign financing and alliances with LEG, further hampers progress (Kypri et al. 2019, Miller et al. 2021). This influence is particularly concerning in Brazil, where private campaign financing has historically created dependence on financiers’ interests (Escola Judiciária do Tribunal Superior Eleitoral 2013). Alcohol industry funding can skew decisions toward industry benefits at the expense of public health.

Our study also found that the alcohol industry perpetuates its economic interests through CSR activities. A systematic review of CSR practices in LAC revealed that over half promoted alcohol products and brands (Pantani et al. 2017). This blurring of CSR and marketing enables promotional activities that evade traditional marketing regulations (Pantani et al. 2017), aligning corporate images with social initiatives while harming public health.

Our findings also suggest that the inertia in regulating alcohol consumption in Brazil may stem from LEG fear of displeasing the population and consequently losing votes. Simultaneously, interview participants noted that some parliamentarians justify their support for the alcohol industry by claiming it contributes to the country’s GDP and generates employment. They also observed that government efforts are largely directed toward combating crack use, relegating alcohol to a secondary priority. Some scholars argue that this emphasis on crack serves as a strategy to obscure deeper social disparities and poverty, addressing the issue superficially while stigmatizing both crack users and the substance itself (Toledo et al. 2017, FIOCRUZ 2022). Meanwhile, alcohol’s economic burden on public health is significant, with an estimated average expenditure of Int$1.17 billion annually from 2010 to 2018 (de Freitas and da Silva 2022).

In our study, the perception that drink-driving regulations are important but undermined by weak enforcement is consistent with findings from other research. An investigation into alcohol use among random drivers in Brazil and Norway, a pioneer in driving under the influence laws with the same blood alcohol concentration limit (<0.2 g/L) as Brazil, found a higher prevalence of drinking and driving in Brazil ([2.7%, 95% confidence interval 2.2–3.3) compared with Norway (0.2%, 95% confidence interval 0.0–0.5) (Gjerde et al. 2014). The authors attributed Norway’s lower prevalence to its intensive enforcement, which includes frequent checkpoints and strict penalties such as license suspension and imprisonment, in contrast to Brazil’s less stringent and, in some cases, bailable sanctions (Brazil 1997).

Adopting regulations without enforcement remains an issue across LAC (Medina-Mora et al. 2021). Despite laws prohibiting alcohol sales to minors (Brasil 2015), 25% of adolescents aged 13–15 years regularly consume alcohol (IBGE 2019). Inadequate enforcement increases the risk of heavy drinking, as seen in open-bar events (Probst et al. 2018, Santos et al. 2023), which facilitate intoxication and serious health risks (Sanchez 2017, Carlini and Sanchez 2018).

Stakeholders disagreed on taxation’s efficacy in reducing consumption. Despite concerns from some participants about supporting tax increases, multiple studies worldwide have demonstrated the public health benefits of raising alcohol prices. Alcohol taxes, whether based on beverage volume, alcohol content, or product value, are widely recognized as one of the most effective and cost-efficient strategies to curb harmful drinking (WHO 2024). These benefits range from direct reductions in consumption to decreases in alcohol-related harms, including cardiovascular disease mortality, reductions in accidents, violence and trauma-related deaths, and road traffic fatalities (Wagenaar et al. 2010, Malawige et al. 2025), as well as declines in non-communicable disease mortality rates (Moyo et al. 2025) and disability-adjusted life years (Hu et al. 2023). Although evidence from other countries in Latin America suggests that price elasticities are smaller in absolute terms, indicating lower sensitivity of consumption to price increases (Paraje et al. 2024), examples from the United Kingdom illustrate that more targeted adjustments, such as substantially increasing taxes on beverages predominantly consumed by high-risk groups (e.g. beer), can yield significantly greater public health gains, including reductions in health inequalities.

Addressing alcohol availability remains crucial. Participants criticized inadequate controls over sales to minors, open-bar parties, and alcohol density in stores. Digital platforms have further eased access, exacerbating consumption. Implementing stricter measures—such as age verification, reducing points of sale, and limiting sales hours—is vital. However, resistance from public interests and industry highlights the need for advocacy and education to align perceptions with public health goals. Cultural and legislative changes are essential to managing alcohol-related issues effectively in Brazil.

Regarding the latest best-buys explored, stakeholders reached a consensus on regulating marketing. Participants likely view this measure as more feasible for Congressional approval since it does not directly limit public access to alcohol, making it less controversial. They highlighted the role of aggressive marketing strategies in normalizing alcohol consumption in Brazil. A 2011 study revealed that advertisements are directly linked to increased consumption, through brand favoritism (odds ratio 5.15) or perceived truthfulness in commercials (odds ratio 2.12) (Faria et al. 2011). This influence is exacerbated by weak enforcement of advertising restrictions and industry self-regulation, allowing widespread marketing across platforms (Vendrame 2017). Interviewees compared this to tobacco advertising restrictions, emphasizing a public health gap (Portes et al. 2018). Strengthened regulations align with global best practices, suggesting that reducing marketing exposure significantly lowers alcohol-related harms.

Although evidence consistently shows that WHO’s best buy policies are far more effective than awareness campaigns (e.g. Alcohol Policy Score) (Pan American Health Organization 2018), political inaction on regulation often leads stakeholders to resort to educational campaigns instead. In the absence of progress on evidence-based measures in Congress, these stakeholders seek to influence societal attitudes toward alcohol by promoting the message that alcohol is harmful. This approach is further reinforced by the cultural acceptance of drinking in Brazil, as campaigns are seen as a more immediate and feasible strategy compared to the long-term effort required for regulatory change. The alcohol industry’s strong influence and the slow pace of legislative processes also discourage advocacy for policy-based solutions.

This study underscores the need for a comprehensive approach to advancing alcohol-related policies in Brazil. This involves not only policy formulation but also fostering a supportive political and cultural environment for implementation. While raising public awareness about alcohol’s health risks is crucial, it is often insufficient alone to change behaviors. Education should include school programs for students and parents, workplace initiatives, and training for healthcare professionals and beverage distributors. Accessible informational materials are also essential. Awareness must be paired with additional measures to generate public demand for policies that mitigate alcohol-related harms.

### Strengths and limitations

This is the first study to explore stakeholder perceptions of alcohol policies in Brazil in detail. By identifying key barriers to implementing evidence-based policies, the study provides practical guidance for policymakers on advancing the topic. A notable strength is its qualitative approach, enabling an in-depth exploration of the cultural, economic, health, and political complexities of Brazil’s alcohol policies. Additionally, the inclusion of diverse representatives, from government officials and LEG to CS members, enriches the analysis with varied perspectives.

Despite its merits, several limitations must be acknowledged. Despite efforts to ensure a diverse sample, the findings may not fully capture the range of opinions on alcohol policies in Brazil. The study’s scope is also limited to the Brazilian context, making comparisons with other settings challenging. The inherent subjectivity of qualitative analysis is another consideration. However, involving all co-authors in the study helped ensure consistency and rigor in the results.

### Further implications for policies

Brazil currently lacks a strategic coalition advocating for effective alcohol policies but could benefit significantly from forming one. Drawing from the Advocacy Coalition Framework (Ritter et al. 2018) and global examples, such a coalition, could drive essential changes in Brazil’s historically resistant alcohol policy landscape. Our study highlights stakeholder agreement on the need for marketing regulations, which could serve as a starting point for collaboration and broader coalition-building over time. Globally, coalitions such as the Global Alcohol Policy Alliance focus on evidence-based actions, including stricter marketing regulations, public education on alcohol harms, and advocacy for healthier policies (Agberotimi and Adekunle 2024). These alliances unite governmental bodies, CS, the private sector, and health experts around shared goals.

In Brazil, a similar coalition could address gaps in alcohol regulation by building trust between experts and NGOs and translating research into actionable communication tailored to the Brazilian context. Such a coalition could provide lawmakers with clear evidence and recommendations, framing alcohol regulation as a public health priority. By delivering concise information on alcohol’s harms and countering industry lobbyists, the coalition could influence policy debates and facilitate overdue regulations.

At the same time, lessons from tobacco control are highly relevant, particularly in Brazil, which has become an international reference in this field. Although progress occurred gradually, beginning in the 1960s, it was during the 1980s that the movement against tobacco gained momentum, driven by the strengthening of national control policies. This process was characterized by the formation of strong coalitions between governmental and non-governmental actors; the adoption of measures grounded in both national and international evidence; the decentralization of actions; and the consolidation of a solid political base. Applying these lessons to alcohol, it is essential for stakeholders to recognize that, although progress may be slow and contested, building and sustaining continuous pressure over time can yield significant public health gains.

## Conclusion

This study highlights that, while respondents generally supported WHO’s alcohol policy ‘best buys,’ doubts about their implementation were evident due to cultural and legislative barriers. Some participants questioned the effectiveness of alcohol price increases in reducing consumption, though this was less common among experienced alcohol experts. These barriers challenge the enforcement of alcohol policies at the population level. In response, some stakeholders have focused on individual interventions as a way to overcome political obstacles and lay the groundwork for future policies. Urgent action is needed to advance alcohol policy in Brazil, including capacity building initiatives and active advocacy coalitions, building on the successful experience of tobacco control. Addressing barriers, enhancing stakeholder skills, and fostering strategic alliances can contribute to successful policy implementation and improved public health.

## Supplementary Material

czaf104_Supplementary_Data

## Data Availability

Data cannot be made available in order to preserve the identities of interviewees.

## Reflexivity statement

This paper reflects a diverse authorship, with four researchers from Latin America and three from Europe. In terms of gender, six authors are cisgender women, and one is a cisgender man. Regarding seniority, the team includes a young PhD lead author, a recent MA graduate, a senior researcher, and professors.
