# Supplementary material for: Untangling the complex web of alcohol policy needs and potential solutions in Brazil: evidence from civil society and political stakeholders
Source: Health Policy Plan. 2025 Dec 18;41(3):365–75. doi: 10.1093/heapol/czaf104 (PMC12972680; doi:10.1093/heapol/czaf104)
Supplement: czaf104_Supplementary_Data [file czaf104_supplementary_data.zip › Supplemental material 1. Coding framework with definitions and interpretive memos..docx]

**Supplemental material 1. Coding framework with definitions and interpretive memos.**

| Theme | Sub-theme | Definitions/memos |
| --- | --- | --- |
| 1. Barriers (to implementing/advancing policies and laws) | 1.1 Alcohol industry | Code any data about how the alcohol industry influences policies (whether through lobbying, exchanging favors or even corruption) and society (through marketing, sports sponsorship); code here any interventions made by the alcohol industry such as campaigns to reduce alcohol consumption in schools or to prevent drunk driving, etc.  **Do not code here barriers that have to do with price, availability, and marketing. Instead, code in item 3.** |
|  | 1.2 Culture/ Normative beliefs | Code here any data on how alcohol is interpreted and incorporated into society; how society interacts with alcohol; the role of alcohol in society; the widespread and frequent use of alcohol in day-to-day social gatherings (such as sports, community, and religious activities), in multiple contexts (such as workplace, streets, bars, clubs, school, homes, etc). |
|  | 1.3 Budget | Code here any data around the lack of funding for new research and/or continuing research, as well as for the development of laws and policies. |
|  | 1.4 Perceptions of alcohol knowledge | Code here data about knowledge on short and long-term individual and social effects of alcohol any data related to the lack of social and political knowledge about public spending arising from the consequences of alcohol consumption, including views on alcohol as a drug/not. |
|  | 1.5 Political/social context | Code here any information about the political context, such as a lack of interest in addressing alcohol-related issues, or political relationships and dynamics (e.g., making decisions to please specific voter groups or organizations). |
| 2. Causes, risk factors, negative consequences of alcohol consumption. | 2.1 Levels of consumption | Code here any data on the prevalence of alcohol consumption in the country, including trends of increase or decrease over time. |
|  | 2.2 Risk factors/causes for alcohol harms | Code here any data on causes associated with alcohol-related harms, as well as information on consumption within specific subgroups (e.g., by gender, age, or other characteristics).  **Data on vulnerable populations should instead be coded under item 2.3.** |
|  | 2.3 Consequences/impact of alcohol/alcohol harms for the general and vulnerable populations | Code here any data on short- and long-term alcohol-related harms to health, society, and the economy (e.g., mortality, acute and chronic diseases, traffic accidents, interpersonal or self-directed violence, excess costs for the public health system, etc.). Include information for both the general population and vulnerable groups (such as Indigenous peoples, LGBTQ individuals, Black populations, women, children, or any other specifically mentioned subgroup). |
|  | 2.4 Illegal alcohol | Code here any data on non-commercial, adulterated, counterfeit, black-market, contaminated, unregistered, or domestically produced alcoholic beverages.  **Mentions of bottle falsification to reduce prices may also be double-coded under ‘Prices.’** |
| 3. Best Buys | 3.1 Prices | Code here any data on prices, taxation, or other pricing strategies (e.g., minimum unit pricing). This may include proposals suggesting that higher prices could reduce alcohol use, or concerns that they could increase the illegal or adulterated alcohol market. |
|  | 3.2 Availability | Code here any data on current alcohol availability (e.g., age limits, local regulations). Also include opinions on proposed changes to availability, such as restrictions on times and places of sale, or on the groups authorized to purchase alcohol (e.g., age limits, perpetrators of violence, individuals with a history of drunk driving, or those who are intoxicated). |
|  | 3.3 Marketing | Code here any data on current alcohol marketing or proposed changes to marketing (e.g., bans on advertising in television, music, or movies), as well as promotions and event sponsorships.  **Do not include opinions on awareness labels; instead, code those under item 4.2.** |
| 4. Policy needs, solutions, and experiences | 4.1 Perception of the current policies | Code here any data on interviewees’ perceptions of current alcohol legislation or policies in the country (e.g., enforcement practices or weaknesses in existing regulations). |
|  | 4.2 Proposed solutions | Code here any data on interviewees’ ideas or suggestions for improving alcohol policies or laws. Include proposed solutions other than the Best Buys. |
|  | 4.3 Experiences of the policy process on alcohol | Code here any experiences related to the process of implementing public policies, including difficulties, gaps, and opportunities. |
|  | 4.4 Experiences on interventions on alcohol | Code here any information about interventions, projects, or actions undertaken by interviewees, including both successful and unsuccessful experiences. |
| 5. Collaboration | 5.1 Current/ past collaborations/working relationships (positive and negative) | Describe existing and past partnerships with other stakeholders, industry, or organizations—whether positive or negative—including how and in what context they collaborate. |
|  | 5.2 Collaborations they would not have | Code here any information about partnerships or collaborations that interviewees explicitly state they would avoid or refuse, and the reasons for this. |
|  | 5.3 Potential collaboration (positive/negative) | Code here any information about potential partnerships with stakeholders, industry, or organizations that interviewees believe would be effective or that they are seeking to establish. |
| 6. Access to and use of alcohol data | 6.1 Use of data | Code here any information on current uses of data sources (e.g., accessing prevalence or patterns of alcohol consumption, or consequences of alcohol use). Also include data that the interviewee has created or collected. |
|  | 6.2 Experiences of data access and use | Code here interviewees’ perceptions about the ease of accessing data, its adequacy, and any issues related to its use. Also include narratives concerning data with potential conflicts of interest involving the alcohol industry. |
|  | 6.3 Data gaps/needs | Code here any data that interviewees would like to have but currently lack or do not use. |
| 7. Other |  | Code here any information of interest that is not covered by the previous codes, such as descriptions of the interviewee’s institution or workplace. No subitems are included at this stage; further classification will be done after the pre-analysis. |
| 8. Interviewee profile/background |  | Code here information about the background or previous occupations of interviewees that may have contributed to their work on alcohol (preferably). |
