# Supplementary material for: Untangling the complex web of alcohol policy needs and potential solutions in Brazil: evidence from civil society and political stakeholders
Source: Health Policy Plan. 2025 Dec 18;41(3):365–75. doi: 10.1093/heapol/czaf104 (PMC12972680; doi:10.1093/heapol/czaf104)
Supplement: czaf104_Supplementary_Data [file czaf104_supplementary_data.zip › Table 1.docx]

**Table 1.** Interviewee characteristics.

| **Participant Number** | **Gender** | **Years of experience in field of alcohol or drugs** | **Type of interviewee/ current field of work (Alcohol/ Tobacco/ Other)** |
| --- | --- | --- | --- |
| **CIVIL SOCIETY (n= 29)** | | | |
| CS02 | Male | 8 | NGO Executive/ NCD risk factor |
| CS03 | Male | +5 | NGO Executive / Alcohol |
| CS07 | Female | +6 | NGO Executive /Tobacco |
| CS08 | Male | 13 | NGO Executive / Substance use |
| CS12 | Male | 17 | NGO Executive/Drink Driving |
| CS13 | Female | 15 | Community Coalition NGO |
| CS14 | Male | +11 | Advocacy for marginalized communities |
| CS15 | Male | +6 | NGO staff/ projects of support for drug users |
| CS17 | Female | 11 | NGO staff/ Harm-reduction |
| CS18 | Female | 0 | Women’s support NGO |
| CS20 | Female | +5 | People with disability NGO |
| CS22 | Female | 25 | NGO staff/ Harm-reduction |
| CS24 | Female | 17 | NGO staff / drug user care |
| C25 | Male | 25 | Addiction treatment NGO |
| CS27 | Male | 24 | Religious NGO |
| **CIVIL SERVANTS, POLICYMAKERS AND**  **LEGISLATORS (n= 31)** | | | |
| PS1 | Female | 1 | Former representative of the Ministry of Health |
| LEG/PS4 | Male | +10 | Prosecutor |
| PS5 | Female | 5-10 | Former representative of the Ministry of Economy |
| LEG/PS6 | Male | 11 | Prosecutor |
| LEG/PS9 | Male | +20 | City councilor |
| PS10 | Female | 17 | Representative of the Ministry of Social Development |
| PS11 | Male | 0 | Former representative of the Ministry of Health |
| PS16 | Male | 0 | Lieutenant |
| PS19 | Male | 20+ | Former representative of the Ministry of Justice |
| LEG21 | Female | 8 | Federal Deputy |
| PS23 | Male | 20+ | Municipal health secretary |
| LEG26 | Male | 0 | City councilor |
| LEG28 | Female | +4 | State Deputy |
| PS29 | Female | +4 | Representative of the National Institute of Cancer |
| PS30 | Male | 4 | Senator's assistant |
| LEG31 | Male | +30 | Federal Deputy |
